# Navigating the Landscape of Molecular Testing and Targeted Treatment of Non–Small Cell Lung Cancer

**DOI:** 10.6004/jadpro.2016.7.3.10

**Published:** 2016-04-01

**Authors:** Sukhmani K. Padda, R. Donald Harvey

**Affiliations:** Stanford University School of Medicine, Palo Alto, California, and Emory University School Medicine, Atlanta, Georgia

Molecular markers and the agents developed to target them have changed the landscape of non–small cell lung cancer (NSCLC) treatment. First-generation targeted agents have been proven to be more effective than chemotherapy in patients with certain driver mutations (i.e., EGFR, ALK) and the newest targeted agents are being developed to address the issue of resistance.

Speakers at JADPRO Live at APSHO emphasized that the use of these targeted treatments begins with tumor tissue, which requires a multidisciplinary approach among oncologists, radiologists, surgeons, pulmonologists, and pathologists. "Sufficient tissue is needed for many reasons," said Sukhmani K. Padda, MD, of Stanford University School of Medicine, Palo Alto, California. "Many tests are performed on this tissue, including confirmation of histologic diagnosis, immunochemistry, and molecular markers."

"Tissue is the issue, but it’s also at a premium," said R. Donald Harvey, PharmD, BCOP, FCCP, FHOPA, of Emory University School of Medicine in Atlanta, Georgia, "meaning you may not be able to go back and get more in some cases."

## GENOMIC DRIVER MUTATIONS

After diagnosis, according to the NCCN Guidelines, management is next stratified by histologic subtype, squamous vs. nonsquamous NSCLC, as the two subtypes lead to different pathways of treatment. "If the patient has nonsquamous histology," said Dr. Harvey, "you need a knee-jerk reaction to send tissue for molecular testing. This is critical, particularly testing for *EGFR* mutations and *ALK* rearrangements, as it can change 1st-line treatment."

Squamous cell NSCLC has a more simplified approach due to lack of targetable driver mutations, Dr. Harvey noted, since first-line therapy uniformly starts with platinum-based therapy. "We know that you should not use bevacizumab (Avastin; for safety concerns), nor should you use pemetrexed (Alimta; for efficacy concerns)."

However, whether the diagnosis is nonsquamous or squamous cell NSCLC, every patient should receive a platinum-based regimen at some point in their treatment course, he added.

## *EGFR*-MUTATED NSCLC

There are three EGFR tyrosine kinase inhibitors (TKIs) approved for the first-line treatment of *EGFR*-mutated NSCLC: afatinib (Gilotrif), erlotinib, and gefitinib (Iressa), which target EGFR activating mutations (i.e., exon 19 deletion and exon 21 L858R). Although erlotinib has approvals in other indications (i.e., maintenance and unselected NSCLC after failure of one chemotherapy), in reality, said Dr. Padda, its most important indication is for the treatment of *EGFR*-mutated NSCLC. Gefitinib and afatinib have narrower indications and are only approved for the treatment of *EGFR*-mutated NSCLC.

As Dr. Padda explained, one of the largest studies with gefitinib was the large Asian IPASS trial (Mok et al., 2009), which was enriched for patients who were never smoking women. At the time, researchers did not know that the *EGFR* mutation was predictive for clinical activity with gefitinib (and other EGFR TKIs), but subsequent analyses clarified that only patients with *EGFR* mutation should receive EGFR TKI therapy first line, and those lacking the *EGFR* mutation should receive platinum-based chemotherapy.

"In the *EGFR*-mutation–negative cohort, the PFS is worse if patients get gefitinib over chemotherapy," she said. "Yet the converse is true: In the *EGFR*-mutation–positive cohort, gefitinib improved PFS over chemotherapy. This landmark study changed the way we began to think about lung cancer."

Multiple studies of treatment-naive *EGFR*-mutated lung cancer have since shown that EGFR TKIs outperform chemotherapy in terms of PFS and response rate. The median PFS for any of these TKIs is between 9 and 12 months. However, Dr. Padda noted, overall survival has not been improved in any of the trials, most likely due to the crossover effect, since the majority of patients with an EGFR mutation eventually received an EGFR TKI (Inoue et al., 2013; Mitsudomi et al., 2010; Zhou et al., 2011; Rosell et al., 2012).

What is also clear is that the subtype of *EGFR* mutation matters. A recent study with afatinib showed that patients with exon 19 deletion do better on EGFR TKI therapy than those with exon 21 L858R, although the mechanism of action still needs to be elucidated (Yang et al., 2015). This enhanced clinical activity in exon 19 deletion patients has also been shown with other EGFR TKIs, at least in relation to improved PFS.

From a pharmacology perspective, there are a few side effects that will be seen in many, if not all patients, Dr. Padda said. Diarrhea is a major side effect and can be self-limiting, but it can be managed with antidiarrheals. Another common side effect is rash, which some patients find "quite debilitating."

## RESISTANCE AT 1 YEAR

Despite existing therapies for *EGFR*-mutated cancer, resistance usually develops at an average of 9 to 12 months. In 60% of cases, the mechanism of resistance to EGFR TKIs is the T790M gatekeeper mutation, a second-site mutation that develops in *EGFR* and prevents the drug from binding as effectively. Although this is the major mechanism of resistance, Dr. Padda noted that other mechanisms are currently being explored.

Clinical trials with third-generation EGFR TKIs targeting T790M show significant promise, including osimertinib (Tagrisso) and rociletinib, with response rates up to 60% (Jänne et al., 2015; Sequist et al., 2015). "These drugs target the original activating mutation and the T790M gatekeeper mutation," said Dr. Padda, "and they’re also designed to spare EGFR wild-type, so patients experience fewer issues with diarrhea and rash." Osimertinib, in fact, was FDA approved soon after JADPRO Live.

According to Dr. Padda, these drugs are now being tested in the 1st-line setting, with response rates over 70% (Ramalingam et al., 2015). "These compounds are now going head-to-head with current 1st-line EGFR TKIs like erlotinib and gefitinib, so we’ll see how they compare to current standard of care."

## *ALK*-REARRANGED NSCLC

For patients with *ALK*-rearranged NSCLC, crizotinib (Xalkori) is the category 1 recommendation in the NCCN guidelines. "This is easy," said Dr. Harvey. "There’s only one drug for the ALK rearrangement in the first line: crizotinib. If the patient progresses, however, that’s where it gets more interesting." If the patient progresses but is asymptomatic, providers can either continue with crizotinib (cautiously) or switch to ceritinib (Zykadia), the 2nd-generation ALK inhibitor, based on the large, phase I ASCEND-1 study (Shaw et al., 2014a; Kim et al., 2016).

"Ceritinib is a harder drug in many ways," said Dr. Harvey. "It has a few more side effects (mostly gastrointestinal) than crizotinib and is a little tougher to handle for patients. However, it’s effective in patients who have failed crizotinib."

Crizotinib also has activity in *ROS1* NSCLC, Dr. Harvey noted, so this is something that should be part of routine molecular panels. With the presence of *ROS1* mutation, crizotinib demonstrated a 72% response rate (Shaw et al., 2014b). Crizotinib received a new indication for this population last month.

Second-generation ALK inhibitors, like ceritinib, have improved CNS activity and many are in development, "Unlike EGFR, where there is a dominant mechanism of resistance, in ALK, the mechanisms of resistance are very heterogeneous," Dr. Harvey explained. "So these second-generation ALK inhibitors are attempting to target a variety of those."

Alectinib (Alecensa), another 2nd-generation ALK inhibitor, was also FDA approved after JADPRO Live. In crizotinib-resistant patients, the ORR to alectinib was 50%, but was higher at 70% among patients without prior chemotherapy (Ou et al., 2015). In ALK inhibitor-naive but not treatment-naive patients, there was very enticing initial preliminary activity with a response rate of 93% (Ohe et al., 2015).

"These drugs are exciting," said Dr. Harvey. "Part of the question will be how to sequence these drugs. Is it best to use crizotinib first, or second? Where will other drugs with clear evidence of activity fit in?"

## References

[1] Inoue A, Kobayashi K, Maemondo M, Sugawara S, Oizumi S, Isobe H, Gemma A, Harada M, Yoshizawa H, Kinoshita I, Fujita Y, Okinaga S, Hirano H, Yoshimori K, Harada T, Saijo Y, Hagiwara K, Morita S, Nukiwa T (2013). Updated overall survival results from a randomized phase III trial comparing gefitinib with carboplatin-paclitaxel for chemo-naïve non-small cell lung cancer with sensitive EGFR gene mutations (NEJ002).. *Annals of oncology : official journal of the European Society for Medical Oncology / ESMO*.

[2] Jänne Pasi A, Yang James Chih-Hsin, Kim Dong-Wan, Planchard David, Ohe Yuichiro, Ramalingam Suresh S, Ahn Myung-Ju, Kim Sang-We, Su Wu-Chou, Horn Leora, Haggstrom Daniel, Felip Enriqueta, Kim Joo-Hang, Frewer Paul, Cantarini Mireille, Brown Kathryn H, Dickinson Paul A, Ghiorghiu Serban, Ranson Malcolm (2015). AZD9291 in EGFR inhibitor-resistant non-small-cell lung cancer.. *The New England journal of medicine*.

[3] Kim Dong-Wan, Mehra Ranee, Tan Daniel S W, Felip Enriqueta, Chow Laura Q M, Camidge D Ross, Vansteenkiste Johan, Sharma Sunil, De Pas Tommaso, Riely Gregory J, Solomon Benjamin J, Wolf Jürgen, Thomas Michael, Schuler Martin, Liu Geoffrey, Santoro Armando, Sutradhar Santosh, Li Siyu, Szczudlo Tomasz, Yovine Alejandro, Shaw Alice T (2016). Activity and safety of ceritinib in patients with ALK-rearranged non-small-cell lung cancer (ASCEND-1): updated results from the multicentre, open-label, phase 1 trial.. *The Lancet. Oncology*.

[4] Mitsudomi Tetsuya, Morita Satoshi, Yatabe Yasushi, Negoro Shunichi, Okamoto Isamu, Tsurutani Junji, Seto Takashi, Satouchi Miyako, Tada Hirohito, Hirashima Tomonori, Asami Kazuhiro, Katakami Nobuyuki, Takada Minoru, Yoshioka Hiroshige, Shibata Kazuhiko, Kudoh Shinzoh, Shimizu Eiji, Saito Hiroshi, Toyooka Shinichi, Nakagawa Kazuhiko, Fukuoka Masahiro (2010). Gefitinib versus cisplatin plus docetaxel in patients with non-small-cell lung cancer harbouring mutations of the epidermal growth factor receptor (WJTOG3405): an open label, randomised phase 3 trial.. *The Lancet. Oncology*.

[5] Mok Tony S, Wu Yi-Long, Thongprasert Sumitra, Yang Chih-Hsin, Chu Da-Tong, Saijo Nagahiro, Sunpaweravong Patrapim, Han Baohui, Margono Benjamin, Ichinose Yukito, Nishiwaki Yutaka, Ohe Yuichiro, Yang Jin-Ji, Chewaskulyong Busyamas, Jiang Haiyi, Duffield Emma L, Watkins Claire L, Armour Alison A, Fukuoka Masahiro (2009). Gefitinib or carboplatin-paclitaxel in pulmonary adenocarcinoma.. *The New England journal of medicine*.

[6] Ohe Y, Nishio M, Kiura K, Seto T, Nakagawa K, Maemondo M, Tamura T (2015). A phase I/II study with a CNS-penetrant, selective ALK inhibitor alectinib in ALK-rearranged non-small cell lung cancer patients: Updates on PFS and safety results from AF-001JP [Abstract 8061].. *Journal of Clinical Oncology (Meeting Abstracts)*.

[7] Ou S-H I, Ahn J S, De Petris L, Govindan R, Yang J C-H, Hughes B, Kim D-W (2015). Efficacy and safety of the ALK inhibitor alectinib in ALK+ non-small-cell lung cancer patients who have failed prior crizotinib: An open-label, single-arm, global phase 2 study (NP28673) [Abstract 8008].. *Journal of Clinical Oncology (Meeting Abstracts)*.

[8] Ramalingam S S, Yang J C, Lee C, Kurata T, Kim D-W, John T, Janne P A (2015). AZD9291, a mutant-selective EGFR inhibitor, as 1st-line treatment for EGFR mutation-positive advanced NSCLC: Results from a phase 1 expansion cohort [Abstract 8000].. *Journal of Clinical Oncology*.

[9] Ramalingam S S, Yang J C, Lee C, Kurata T, Kim D, John T, Janne P A (2015). AZD9291 in treatment-naïve EGFRm advanced NSCLC: AURA 1st-line cohort [Abstract MINI16.07].. *International Association for the Study of Lung Cancer 16th WCLC*.

[10] Rosell Rafael, Carcereny Enric, Gervais Radj, Vergnenegre Alain, Massuti Bartomeu, Felip Enriqueta, Palmero Ramon, Garcia-Gomez Ramon, Pallares Cinta, Sanchez Jose Miguel, Porta Rut, Cobo Manuel, Garrido Pilar, Longo Flavia, Moran Teresa, Insa Amelia, De Marinis Filippo, Corre Romain, Bover Isabel, Illiano Alfonso, Dansin Eric, de Castro Javier, Milella Michele, Reguart Noemi, Altavilla Giuseppe, Jimenez Ulpiano, Provencio Mariano, Moreno Miguel Angel, Terrasa Josefa, Muñoz-Langa Jose, Valdivia Javier, Isla Dolores, Domine Manuel, Molinier Olivier, Mazieres Julien, Baize Nathalie, Garcia-Campelo Rosario, Robinet Gilles, Rodriguez-Abreu Delvys, Lopez-Vivanco Guillermo, Gebbia Vittorio, Ferrera-Delgado Lioba, Bombaron Pierre, Bernabe Reyes, Bearz Alessandra, Artal Angel, Cortesi Enrico, Rolfo Christian, Sanchez-Ronco Maria, Drozdowskyj Ana, Queralt Cristina, de Aguirre Itziar, Ramirez Jose Luis, Sanchez Jose Javier, Molina Miguel Angel, Taron Miquel, Paz-Ares Luis (2012). Erlotinib versus standard chemotherapy as first-line treatment for European patients with advanced EGFR mutation-positive non-small-cell lung cancer (EURTAC): a multicentre, open-label, randomised phase 3 trial.. *The Lancet. Oncology*.

[11] Sequist Lecia V, Soria Jean-Charles, Goldman Jonathan W, Wakelee Heather A, Gadgeel Shirish M, Varga Andrea, Papadimitrakopoulou Vassiliki, Solomon Benjamin J, Oxnard Geoffrey R, Dziadziuszko Rafal, Aisner Dara L, Doebele Robert C, Galasso Cathy, Garon Edward B, Heist Rebecca S, Logan Jennifer, Neal Joel W, Mendenhall Melody A, Nichols Suzanne, Piotrowska Zofia, Wozniak Antoinette J, Raponi Mitch, Karlovich Chris A, Jaw-Tsai Sarah, Isaacson Jeffrey, Despain Darrin, Matheny Shannon L, Rolfe Lindsey, Allen Andrew R, Camidge D Ross (2015). Rociletinib in EGFR-mutated non-small-cell lung cancer.. *The New England journal of medicine*.

[12] Shaw Alice T, Kim Dong-Wan, Mehra Ranee, Tan Daniel S W, Felip Enriqueta, Chow Laura Q M, Camidge D Ross, Vansteenkiste Johan, Sharma Sunil, De Pas Tommaso, Riely Gregory J, Solomon Benjamin J, Wolf Juergen, Thomas Michael, Schuler Martin, Liu Geoffrey, Santoro Armando, Lau Yvonne Y, Goldwasser Meredith, Boral Anthony L, Engelman Jeffrey A (2014). Ceritinib in ALK-rearranged non-small-cell lung cancer.. *The New England journal of medicine*.

[13] Shaw Alice T, Ou Sai-Hong I, Bang Yung-Jue, Camidge D Ross, Solomon Benjamin J, Salgia Ravi, Riely Gregory J, Varella-Garcia Marileila, Shapiro Geoffrey I, Costa Daniel B, Doebele Robert C, Le Long Phi, Zheng Zongli, Tan Weiwei, Stephenson Patricia, Shreeve S Martin, Tye Lesley M, Christensen James G, Wilner Keith D, Clark Jeffrey W, Iafrate A John (2014). Crizotinib in ROS1-rearranged non-small-cell lung cancer.. *The New England journal of medicine*.

[14] Yang James Chih-Hsin, Wu Yi-Long, Schuler Martin, Sebastian Martin, Popat Sanjay, Yamamoto Nobuyuki, Zhou Caicun, Hu Cheng-Ping, O'Byrne Kenneth, Feng Jifeng, Lu Shun, Huang Yunchao, Geater Sarayut L, Lee Kye Young, Tsai Chun-Ming, Gorbunova Vera, Hirsh Vera, Bennouna Jaafar, Orlov Sergey, Mok Tony, Boyer Michael, Su Wu-Chou, Lee Ki Hyeong, Kato Terufumi, Massey Dan, Shahidi Mehdi, Zazulina Victoria, Sequist Lecia V (2015). Afatinib versus cisplatin-based chemotherapy for EGFR mutation-positive lung adenocarcinoma (LUX-Lung 3 and LUX-Lung 6): analysis of overall survival data from two randomised, phase 3 trials.. *The Lancet. Oncology*.

[15] Zhou Caicun, Wu Yi-Long, Chen Gongyan, Feng Jifeng, Liu Xiao-Qing, Wang Changli, Zhang Shucai, Wang Jie, Zhou Songwen, Ren Shengxiang, Lu Shun, Zhang Li, Hu Chengping, Hu Chunhong, Luo Yi, Chen Lei, Ye Ming, Huang Jianan, Zhi Xiuyi, Zhang Yiping, Xiu Qingyu, Ma Jun, Zhang Li, You Changxuan (2011). Erlotinib versus chemotherapy as first-line treatment for patients with advanced EGFR mutation-positive non-small-cell lung cancer (OPTIMAL, CTONG-0802): a multicentre, open-label, randomised, phase 3 study.. *The Lancet. Oncology*.

